# Complete Genome Sequences of Two Phage-Like Plasmids Carrying the CTX-M-15 Extended-Spectrum β-Lactamase Gene

**DOI:** 10.1128/genomeA.00102-17

**Published:** 2017-05-11

**Authors:** Anna Colavecchio, Julie Jeukens, Luca Freschi, Jean-Guillaume Edmond Rheault, Irena Kukavica-Ibrulj, Roger C. Levesque, Jeffrey LeJeune, Lawrence Goodridge

**Affiliations:** a Department of Food Science and Agricultural Chemistry, Food Safety and Quality Program, McGill University, Ste-Anne-de-Bellevue, Canada; b Institut de Biologie Intégrative et des Systèmes (IBIS), Université Laval, Québec, Canada; c Food Animal Health Research Program, The Ohio State University, Wooster, Ohio, USA

## Abstract

Two similar phage-like plasmids carrying CTX-M-15 resistance cassettes were identified from two environmental *Escherichia coli* isolates. They demonstrate strong nucleotide sequence identity to the phage-like plasmid pECOH89 and *Salmonella* bacteriophage SSU5.

## GENOME ANNOUNCEMENT

β-Lactam antibiotics are the most common treatment for bacterial infections and continue to promote the emergence of extended-spectrum β-lactamase (ESBL) resistance in Gram-negative bacteria worldwide (1). Among the various ESBL enzymes, the rapid emergence of CTX-M variants has been referred to as a pandemic (2). Multiple factors contribute to the spread of antibiotic resistance, including the involvement of bacteriophages (3–5).

Two environmental *Escherichia coli* strains isolated from the feces of wildlife closely associated with bovine feedlots in Colorado, USA, were found to contain phage-like plasmids harboring CTX-M-15. We performed whole-genome sequencing of the two *E. coli* isolates to determine the nature of the phage-like plasmids.

Whole-genome sequencing was performed at the EcoGenomics analysis platform (Ibis; Université Laval, Québec, Canada) on an Illumina MiSeq using 300-bp paired-end libraries with 30× coverage. The raw reads were assembled using the A5 pipeline (6). PHASTER (7) and PhiSpy (8) identified prophage regions. ResFinder (9) identified antibiotic resistance genes within these prophage regions. RAST (10) annotated the phage-like plasmid, and tRNAscan (11) identified tRNA genes. ACLAME (12) identified the mobile genetic element neighboring the CTX-M-15 gene.

The complete sequences of the phage-like plasmids AnCo1 from *E. coli* 243 and AnCo2 from *E. coli* 244 are 112,210 bp with a G+C content of 46.1%, and 109,071 bp with a G+C content of 46.3%, respectively. AnCo1 has 134 coding sequences (CDSs) and 2 tRNAs for asparagine and threonine, while AnCo2 harbors 132 CDSs and no tRNA genes. Both phage-like plasmids are highly homologous, since they share 127 CDSs with 100% nucleotide sequence identity. AnCo1 encodes seven additional CDSs in comparison with AnCo2, which mainly encodes hypothetical proteins.

Genomic analysis revealed that AnCo1 and AnCo2 share 122 CDSs and 117 CDSs, respectively, with >91% nucleotide sequence identity to 128 CDSs of a recently published phage-like plasmid pECOH89 isolated from *E. coli* strain H89 (Genbank accession no. HG530657.1). The phage-like plasmids carry a CTX-M-15 cassette consisting also of a hypothetical protein and a mobile genetic element identified as an ISE*cp1* transposase gene. The CTX-M-15 cassette has 100% nucleotide sequence identity to pECOH89, except for the absence of the putative *orf477*. Nucleotide sequence identity was also detected to the virulent *Salmonella*-specific bacteriophage SSU5 (GenBank accession no. JQ965645). In total, AnCo1 and AnCo2 share 92 CDSs and 91 CDSs out of 130 CDSs of SSU5, respectively, with >68% nucleotide sequence identity. The phage-like plasmids have >78% nucleotide sequence identity to the phage genes of SSU5 which encode phage tail proteins, a phage tail fiber protein, the terminase large subunit, the major capsid protein, the tape measure protein, a holin, and an endolysin.

In conclusion, we present two novel and similar phage-like plasmids carrying a CTX-M-15 resistance cassette and isolated from environmental *E. coli*. We suggest that these two entities are phage-like plasmids of which their phage region has nucleotide sequence identity to *Salmonella* phage SSU5.

### Accession number(s).

The complete genome sequences of AnCo1 and AnCo2 have been deposited in GenBank under the accession numbers KY515224 and KY515225, respectively.
